# Exploring Antioxidant Activity, Organic Acid, and Phenolic Composition in Strawberry Tree Fruits (*Arbutus unedo* L.) Growing in Morocco

**DOI:** 10.3390/plants9121677

**Published:** 2020-11-30

**Authors:** Hafida Zitouni, Lahcen Hssaini, Rachida Ouaabou, Manuel Viuda-Martos, Francisca Hernández, Sezai Ercisli, Said Ennahli, Zerhoune Messaoudi, Hafida Hanine

**Affiliations:** 1 Laboratory of Bioprocess and Bio-interfaces, Faculty of Science and Technics, University Sultan Moulay Slimane, BO 523, Beni-Mellal 23000, Morocco; hafida2012ziitn@gmail.com; 2 Research Unit of Plant Breeding and Plant Genetic Resources Conservation, National Institute for Agricultural Research (INRA), BO 578, Meknes 50000, Morocco; hssaiini@gmail.com; 3 LICVEDDE/ERIDDECV (Research Team of Innovation and Sustainable Development & Expertise in Green Chemistry), Faculty of Science Semlalia, Cadi Ayyad University, Marrakesh 40000, Morocco; rachaouaabou@gmail.com; 4 Departamento de Tecnología Agroalimentaria, Tecnología Agroalimentaria, IPOA, Escuela Politécnica Superior de Orihuela, (Universidad Miguel Hernández), Ctra Beniel, km 3.2, E−03312 Orihuela, Spain; mviuda@umh.es; 5 Grupo de Investigación de Producción Vegetal y Tecnología, Departamento de Producción Vegetal y Microbiología, Producción Vegetal y Microbiología, Cuela Politécnica Superior de Orihuela (Universidad Miguel Hernández de Elche), Ctra. de Beniel, km 30.2, E−03312 Orihuela, Spain; francisca.hernandez@umh.es; 6 Department of Horticulture, Agricultural Faculty, Ataturk University, 25240 Erzurum, Turkey; sercisli@gmail.com; 7 Department of Arboriculture, Horticulture and Viticulture, National School of Agriculture, (ENA), BO S/40, Meknes 50000, Morocco; ennahlisaid@gmail.com (S.E.); messaoudiz@yahoo.fr (Z.M.)

**Keywords:** *Arbutus unedo* L, antioxidant activity, organic acid, polyphenolic profiles

## Abstract

This study aimed to explore the main biochemical components and the antioxidant capacity of five strawberry tree fruits using three antioxidant essays within the ecotypic comparison scheme, to find out the most valuable fruit presenting disease-preventing properties. Total phenols, total flavonoids, total anthocyanins, antioxidant activity (DPPH, ABTS, and β-Carotene bleaching assays), pH, titratable acidity, soluble solids, and moisture content were investigated in five strawberry tree genotypes belonging to several areas in Morocco. Phenolic compounds were also identified using high performance chromatography (HPLC), with a diode array detector (DAD). High significant differences (*p* ˂ 0.05) were revealed among the examined genotypes regarding their total phenols (25.37–39.06 mg gallic acid equivalents (GAE)/g Dry weight (DW), total flavonoids (3.30–7.07 mg RE/g Dry weight (DW), total anthocyanins (0.15–0.64 mg cya-3-glu/100g Dry weight (DW), pH (2.44–3.92), titratable acidity (0.65–1.01 g malic acid/100g Fresh weight (FW), and soluble solids (14.83–18.53%). The average radical scavenging capacity, assessed using three methods, exhibited the following concentration ranges: 3.33–21.08, 2.25–19.58, and 1.08–13 mg Ascorbic Equivalent (AAE/g Dry weight(DW) for the DPPH scavenging test, ABTS, and β-carotene bleaching, respectively. Seventeen phenolic compounds were identified in sampled cultivars. Gallocatechol and catechin were found to be the major phenolic compounds. The correlation matrix revealed significant correlations among investigated variables, particularly ABTS and DPPH. The principal component analysis showed that the first three components formed 90.25% of the total variance. The following variables: chlorogenic acid, ellagic acid derivative, ellagic acid, rutin, and cyanidin−30.5-diglucoside, were the most involved in the total variance. The results revealed highly promising physico-biochemical profiles within the studied strawberry tree genotypes.

## 1. Introduction

Fruit trees present a widely genetic diversity reflected in their broad range of mopho-agronomic, multiple pharmacological activities, and biochemical composition, which are, accordingly, very diverse. Their fruits are fundamentally very rich in terms of bioactive molecules, including phytochemicals (phenolics, carotenoids, lignans, stilbenes, etc.), vitamins (mainly vitamins, A, C, E, and K), minerals (i.e., potassium, calcium, and magnesium), and dietary fibers, which have vital functions in human health by alleviating several chronic diseases [1,2,3,4]. 

Fruits (berries in particular) as a source of nutrients and bioactive molecules and health-promoting properties, remains, so far, a hot topic in the scientific community. Epidemiological reports have consistently shown sufficient evidence proving that the regular consumption of berries is directly linked to the prevention of coronary diseases. The antioxidant attributes of these compounds act as reducing agents, metal chelators, hydrogen donors, and singlet oxygen quenchers [2,3,4].

The strawberry tree (*A. unedo*) is generally considered a small tree, usually smaller than 4 m. During autumn, it bears orange colored fruit, naturally grown as a population or solitary tree in Mediterranean countries, such as Morocco, Tunisia, Algeria, Turkey, Syria, Greece, Croatia, France, Portugal, and Spain [5]. The strawberry tree is recognized as a medicinal species, with high antioxidant potential, due mainly to polyphenols concentrated in its fruit, which play a major role in safeguarding health, because of their biological functions, such as antimutagenicity, anticarcinogenicity, and antiaging [6]. Strawberry tree fruit, being a red spherical berry, is not only suitable for the production of alcoholic beverages, jams, jellies, and marmalades [7], but also for medicinal purposes [8]. 

In Morocco—a hotspot of fruit tree diversity—*A. unedo* is known as “Sasnou”, and it is widely used by the local population in traditional medicine as antiseptics, diuretics, laxatives, and, more recently, used in therapy for diabetes and arterial hypertension [8,9]. The latter has been demonstrated in previous reports (the total tannin concentration of the leaf extract produces an in vitro inhibition of platelet aggregation) [10,11]. Both fruits and leaves have been used for medicinal purposes for centuries, as they possess good antimicrobial and antioxidant proprieties. Furthermore, strawberry tree fruits are well-known as a good dietary source of antioxidants, including phenolic compounds (e.g., anthocyanins and other flavonoids, gallic acid derivatives, and tannins), vitamins C and E, and carotenoids [7,8,12,13,14,15,16,17]. These bioactive plant secondary metabolites are systematically involved in the species’ biological systems, such as pigmentation, growth, reproduction mechanisms, protection against predators, etc. [14]. Moreover, they have been used since ancient times as primary and supplemental treatments for various ailments, supporting normal physiological functions [18]. Phenolic compounds can amplify the human defense system to eliminate cancer cells and block angiogenesis, which is the formation of new blood vessels, essential for tumor development [19]. Recently, several studies have shown that strawberry antioxidants and bioactive compound amounts strongly depend on genetic background. Moreover, geographical origin has significant influence on the biosynthesis of these nutriments during ripening [20,21,22].

More recently, there has been an increased interest in using naturally occurring phytochemicals from novel, raw material, for the prevention and treatment of different chronic human diseases [23,24,25,26,27]. Among phytochemicals, phenolics from a large number of fruits and beverages have been shown to prevent cancer and cardiovascular diseases [28]. Strawberry tree fruit is also a rich source of phytochemicals. Previous phytochemical studies on the plant showed the presence of three anthocyanins: delphinidin 3-*O*-galactoside, cyanidin 3-*O*-galactoglucoside, and cyanidin 3-*O*-galactoside [29]. *Arbutus unedo L.* fruits were reported very high when compared with 27 of the other fruits [30]. 

Despite the high popularity of these wild fruits in Morocco, the diversity within the species is still largely unknown. Moreover, data regarding a complete ethnomedicinal and nutritional assessment of *A. unedo* fruits are missing. The high nutritional quality and bioactive compounds of these berries are likely to be lost if not documented. Therefore, this species remains, so far, underexploited, due to the lack of awareness of their potential, market demand, and value addition. However, to date, the genetic resources of *A. unedo* still face a serious threat of extinction, mainly because of climate change and extensive urbanization. To preserve the existing diversity within the species, scientific survey, core-collection building, and large-scale assessment are urgently required to ensure food and nutritional security of rural populations and to achieve sustainable development. 

Biochemical markers have been widely used in breeding studies and in investigations into diversity of species, and the relationship between genotypes and their wild relatives. More recently, biochemical content, in particular, bioactive content of fruits, has been widely researched, in terms of their human health benefits. Scientists are now searching to find genotypes that can meet farmers’ and industrial requirements, regarding their agronomic and functional properties, in order to use them in breeding programs to develop new chemotypes that hold high nutritional proprieties, making them suitable ingredients for the food industry and for health applications [31]. However, in Morocco, very few studies have been devoted toward strawberry tree fruits. Thus, the objective of this study was to investigate, for the first time, strawberry tree fruits in terms of their main physical and biochemical characteristics, in a comparative scheme of five prospected Moroccan clones. The main purposes of this work are: (i) to assess the quality of strawberry tree fruits (pH, titratable acidity, soluble solid); (ii) to evaluate the polyphenolic profiles and antioxidant activities of strawberry tree fruits using three methods (DPPH, ABTS, and beta-carotene bleaching assays); (iii) to identify the correlations among all studied parameters in order to determine the ones that are potentially important in assessing strawberry tree genotypes; and (iv) to evaluate the biochemical diversity among the strawberry tree genotypes belonging to several Moroccan geographical origins. Herein, we intend to present a complete database regarding biochemical composition and antioxidant properties of these cultivars in order to valorize them as an invaluable source of nutrients and nutraceuticals. This will facilitate the breeding and selection of new strawberry tress cultivars by integrating the scattered desired attributes.

## 2. Materials and Methods 

### 2.1. Plant Material

**Figure 1:**
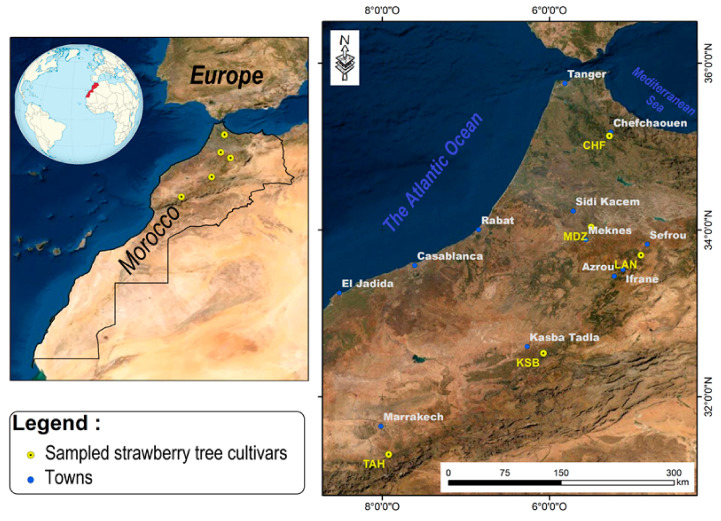
Sampling points and geographic origin geographic of the strawberry tree genotypes studied.

Five genotypes of the strawberry tree (*Arbutus unedo* L.) were collected between October and November 2019 named (Chefchaouen (CHF), Moulay Driss Zerhoun (MDZ), Laanoucer (LAN), El Ksiba (KSB), and Thnaout (TAH)), where they grew spontaneously (Table 1; Figure 1). Morphological key characters of both tree and its flowers and leaves were used for in-situ trees selection (International Plant Genetic Resources Institutes (IPGRI)) and CIHEAM) [32]. This selection was also performed through a survey with a local population, since surveyed geographical sites represent an endemic area for species growth in Morocco. Each area hosts typical, spontaneously growing strawberry tree clones.

The strawberry tree cultivars, herein examined, presented round-shaped fruits, with an index varying from 0.916 to 1.001, and weight ranging between 1.32 and 4.46 g. The cluster length ranged from 32.64 to 74.01, while the number of fruits per cluster varied from 2.71 to 5.34. Moreover, leaf weight was in the range of 0.246 to 0.486 g. Likewise, the flower cluster length varied from 7.15 to 13.41. The investigated cultivars displayed significant differences based on their fruits, flowers, and leaf morphology.

At each geographical location, random fruits, having uniform size and maturity, with no diseases and visual blemishes, were harvested at their fully ripened stage, and transferred to the laboratory for biochemical and phytochemical analysis. Fruits picked at different positions around the canopy were considered fully ripened when their color turned from yellow/green to red, and when they were easily separated from the twig. Fruits were frozen at −80 °C, freeze-dried, and ground, then kept in appropriate conditions for subsequent use.

### 2.2. UV-VIS Profile Determination

The bioactive constituents of the sample extracts were scanned in a wavelength ranging from 340–800 nm by using a UV-VIS spectrophotometer (spectrophotometer Spectra Physics JASCO V730, instrument, JASCO corporation 2967-5 Ishikawa-matchi Hachioji-shi, Tokyo 192-8537, Japan), and the main absorbance peaks were identified.

### 2.3. Biochemical Analyses

Total soluble solids (TSS) were assessed according to Association of Official Analytical Chemists (AOAC) [33] with a digital refractometer (Atago N1; Atago Co. Ltd., Tokyo, Japan), at room temperature, and expressed as Brix. Total titratable acidity (TA) was assessed according to AOAC [33] using an automatic titration device (877 Titrino plus, Metrohm ion analyses CH9101, Herisau, Switzerland), 0.1 N NaOH up to pH 8.1, using 1 mL diluted juice in 25 mL distilled H_2_O. Results were expressed as g of malic acid per 100 g FW. The pH was measured using a pH meter, according to the method described by AOAC [33]. 

### 2.4. Organic Acids and Ascorbic Acid Profiles

A total of 0.5 g of each sample was extracted with 5 mL of Milli-Q water by incubation for 30 min under ultra-sonication. The mixture was then centrifuged at 150,000× *g* for 20 min (Sigma 3–18 K; Sigma, Laborzentrifugen GmbH, Osterode am Harz, Germany). The supernatant was filtered using a 0.45 μm Millipore filter and immediately used for analysis. All extractions were carried out in triplicate.

The chromatographic analysis was performed as reported by Garcia-Salas et al. [34]. Briefly, 10 μL of each extract were injected into a Hewlett-Packard HPLC Series 1100 (Wilmington, DE, USA) using an autosampler. The UV detector was set at 210 nm and coupled with a refractive index detector (HP 1100, G1362A). A column (Supelcogel TM C−610H column 30 cm × 7.8 mm) and apre-column (Supelguard 5 cm × 4.6 mm; Supelco, Bellefonte, PA, USA) were used for the analyses of both organic acids and ascorbic acid. The elution buffer consisted of 0.1% phosphoric (*v/v*) at a flow rate of 0.5 mL min^−1^. Organic acids were measured at a wavelength of 210 nm using a diode-array detector (DAD). Analysis were performed in triplicated and results were expressed as g 100 g^−1^ of dry weight (DW).

### 2.5. Phytochemical Composition

#### 2.5.1. Extraction Procedure 

One g of powder from each sample was mixed with 25 mL of ethanol (1:25, *w/v*) at 25 °C for 15 min using an IKA T−18 digital Ultra-Turrax homogenizer. The homogenate was then centrifuged for 10 min at 6000 rpm and the supernatant was removed from the residue. The latter was homogenized and the supernatant removed as above. The supernatants were then combined and filtered. 

#### 2.5.2. Total Phenols (TP)

Phenolic contents (PT) of sampled strawberry tree fruits were determined, according to Ben Salem et al. [35]. Moreover, 100 µL of diluted sample (1/100) with ethanol was added to 400 µL of 1/10 diluted Folin Ciocalteu reagent. After 5 min, 500 µL of 10% (*w/v*) sodium carbonate solution was added. After 1 h of incubation at room temperature, absorbance at 765 nm (spectrophotometer Spectra Physics JASCO V730, corporation 2967-5 Ishikawa-matchi Hachioji-shi, Tokyo 192-8537, Japan) was measured in triplicate. Total polyphenols content (TP) was expressed as milligrams gallic acid equivalents (GAE) per g dry weight of strawberry tree fruit.

#### 2.5.3. Total Flavonoids (TF)

Total flavonoids of samples were determined, as described by Lamaison and Carnat [36]. Moreover, 1 mL of the sample was mixed with 1 mL of a 2% aluminum chloride solution. The mixture was incubated at room temperature for 15 min. The absorbance was measured at 430 nm (with a spectrophotometer Spectra Physics JASCO V730, Japan). The results were expressed as rutin equivalent per dry weight of strawberry tree fruit.

#### 2.5.4. Total Anthocyanins 

Total anthocyanins content (TAC) of samples were determined using the pH differential method with some modifications, according to Giusti and Wrolstad [37]. A 1 mL aliquot of each sample extract was mixed with 980 μL of KCl buffer (pH1.0) and NaOAc buffer (pH 4.5). After the mixtures were incubated at a room temperature for 15 min, the absorbance was read at 510 nm and 700 nm for both sets of pH 1.0 and 4.5 solutions. Total anthocyanins were estimated using Equation (1), and their concentrations were expressed as milligrams of cyanidin-3-glucoside equivalents in 100 g of DW.

TA = (A*MW*DF *1000/Ɛ*L) (1)

where, *A*: absorbance = (A510 nm–A700 nm) _pH1.0_—(A510 nm–A700 nm) _pH4.5_; *MW*: molecular weight (449.2 g/mol); *DF*: dilution factor; *Ɛ*: molar absorptivity coefficient of cyanidin-3-glucoside (260.900 L/mol cm).

### 2.6. Antioxidant Activities 

The antioxidant activities were determined using three different assays: (i) DPPH assay, (ii) ABTS assay, and (iii) the β-Carotene bleaching test. Each essay was performed in triplicate, using Lambda EZ 150 (Spectra Physics JASCO V730, Japan) spectrophotometer. For all essays, a calibration curve within a range of 0.5−5.0 mg of ascorbic acid g^−1^ was used for the quantification of the three methods, showing good linearity (R2 ≥ 0.998). Results were expressed accordingly as mg ascorbic acid equivalent (AAE) per dry weight.

#### 2.6.1. DPPH Free Radical-Scavenging Capacity 

The DPPH (10.1-diphenyl−2-picrylhydrazyl) radical scavenging activity of the samples was determined according to Ben Salem et al. [35]. Thus, DPPH solution was prepared by dissolving 0.1 g of DPPH in 1 L methanol (HPLC quality). Then, to each extract (125 µL), 1 mL of this solution was added. The mixture was stirred thoroughly and incubated in the dark at room temperature for 10 min. A control solution was prepared by adding equal volumes of DPPH and methanol. The absorbance of both sample and control was measured at 517 nm, and their scavenging activity of DPPH radicals was determined using the following equation: DPPH scavenging activity % inhibition).

DPPH scavenged (%) = {(Ac − As)/Ac} * 100(2)

where, Ac and AS refer to the control and sample absorbances, respectively.

IC50 value (mg extract/g DW) defines the inhibitory concentration at which tested radicals were scavenged by 50%. It was calculated by plotting inhibition percentage of each test against the sample extract dilutions.

#### 2.6.2. ABTS Assay

The ABTS (20.2-azinobis- (3-ethylbenzothiazoline−6-sulphonic acid)) radical scavenging assay was performed according to Dorman and Hiltunen. [38]. Thus, 990 µL of each sample extract was incubated in 10 µL ABTS (7 mM)-ETOH and 2.45 mM potassium persulfate solution after sonicated at 20 °C for 15 min during 16 h in the dark. The mixtures were the incubated for 18 h in the darkness at room temperature. The ethanol (HPLC quality) was used to dilute the stock solution of ABTS until absorbance of 0.70 ± 0.05 was reached at a wavelength of 734 nm. 

#### 2.6.3. β-Carotene Bleaching Assay

The Beta-carotene blanching essay was carried out according to Barros et al. [39]. β-carotene (0.5 mg) in 1 mL of chloroform was taken in an amber bottle and mixed with 200 mg of linolenic acid and 600 mg of Tween 80 (polyoxyethylene sorbitan monopalmitate). The chloroform was removed under nitrogen, and the resulting solution was immediately diluted with 30 mL of triple distilled water; the emulsion was mixed well for 1 min. The emulsion was further diluted with 120 mL of oxygenated water and used for assay. To each sample extract (0.5 mL), 2.5 mL of the prepared emulsion mixture was added and then vigorously mixed. A control consisting 0.5 mL of ethanol and 2.5 mL of emulsion was also analyzed. The absorbance of reaction mixture was read immediately (t = 0) at 470 nm against blank, consisting of emulsion mixture, except β-carotene, and at the 60 min interval for 2 h (t = 120). The tubes were incubated in a water bath at a temperature of 50 °C between measurements. Color measurement was monitored until the β-carotene color disappeared. The linoleic acid peroxidation inhibition uses the following Equation (3):

AA = 100 [1 − (A_o_ − A_t_)/(A_00_ − A_ot_)] (3)

where, A_o_ and A_oo_ refer to the absorbance measured at the beginning of samples and control incubation, respectively. A_t_ and A_ot_ are the final absorbance of samples and control, respectively. 

### 2.7. Extraction and Determination of Polyphenolic Compounds

#### 2.7.1. Extraction Method

Samples (1 g) were mixed with 10 mL of methanol:water (80:20, *v/v*); the mixtures were then sonicated for 30 min and macerated for one hour in refrigeration (4 °C). The samples were then centrifuged for 10 min, 8000× *g* at 4 °C. The supernatants were collected and the pellets were mixed with 10 mL of acetone:water (70:30, *v/v*), and the same steps were repeated (sonication, maceration, and centrifugation). Then, the supernatants were combined and evaporated to dryness using a rotary evaporator R−205 under reduced pressure, at 40 °C. Moreover, 5 mL of methanol were added to the residue, and the mixture was well-shaken in a Vortex for 2 min. Due to the high sugar content present in the samples, which could interfere with the HPLC column, the samples were loaded onto a C18 Sep-Pak cartridge, previously conditioned with 5 mL of methanol, 5 mL of pure water, and then, with 5 mL of 0.01 mol/L HCl. The cartridge was washed with 5 mL of pure water and then eluted with acidified methanol (0.1 g/L HCl). The collected fractions were stored at −20 °C until further use. 

#### 2.7.2. Determination of Polyphenolic Profiles

Polyphenolic profiles of all samples were determined according to Genskowsky [40]. A volume of 20 µL of the samples were injected into a Hewlett-Packard HPLC series 1200 instrument equipped with a diode array detector (DAD) and a C18 column (Mediterranean sea 18, 25 × 0.4 cm, 5 cm particle size) from Teknokroma, (Barcelona, Spain). This spectrophotometer uses a Xenon lamp, illuminant D65, 10° observer, SCI mode, 11 mm aperture of the instrument for illumination and 8 mm for measurement. Polyphenolic compounds were analyzed in standard and sample solutions, using a gradient elution at 1 mL/min. The mobile phases were composed by formic acid in water (1:99, *v/v*) as solvent A and acetonitrile as solvent B. The chromatograms were recorded at 280, 320, 360, and 520 nm. Polyphenolic compound identification was carried out by comparing UV absorption spectra and retention times of each compound with those of pure standards injected in the same conditions.

### 2.8. Statistical Analysis

All analyses were performed in triplicate. The means were evaluated according to descriptive statistics represented as mean ± SE. Data analysis was performed using IBM SPSS v22. Analysis of variance (ANOVA) was performed to test significant differences among the samples. The differences in studied variables were estimated with Duncan new multiple range (DMRT) test. Correlation coefficients and their levels of significance were calculated using the Pearson correlation. Principal Component Analysis (PCA) was carried out using correlation matrix to achieve a better understanding of the trends and relationships among investigated biochemical variables. Moreover, it served to determine the main factors to reduce the number of effective parameters to use in decimating the sampled genotypes. In addition, a scatter plot was created according to the first three principal components (PC1, PC2, and PC3). A two-dimensional hierarchical clustered heatmap was applied to the dataset using R software 3.0.2. Prior to these analyses, data were standardized to a comparable scale (µ = 0 and σ = 1). In this presentation of data, the effect size measure is represented by the color intensity. The heatmap groups similar rows and columns together, with their similarity represented by a dendrogram. 

With the advent of high-throughput experiments, biochemical attributes have often been coupled with chemometrics to extract features relevant for better understanding of multiple associations between these attributes. For this purpose, heatmap and PCA are the two popular methods for analyzing this type of data. In this study, both methods were used to investigate the change of investigated metabolites of sampled cultivars from different geographical origins. These methods are of importance to achieve better understanding of complex biological systems, where one-way direction is assumed [41]. They also aim to explore the associations between these factors with regards to the genetic factor.

## 3. Results and Discussion

### 3.1. UV-VIS Profile 

**Figure 2:**
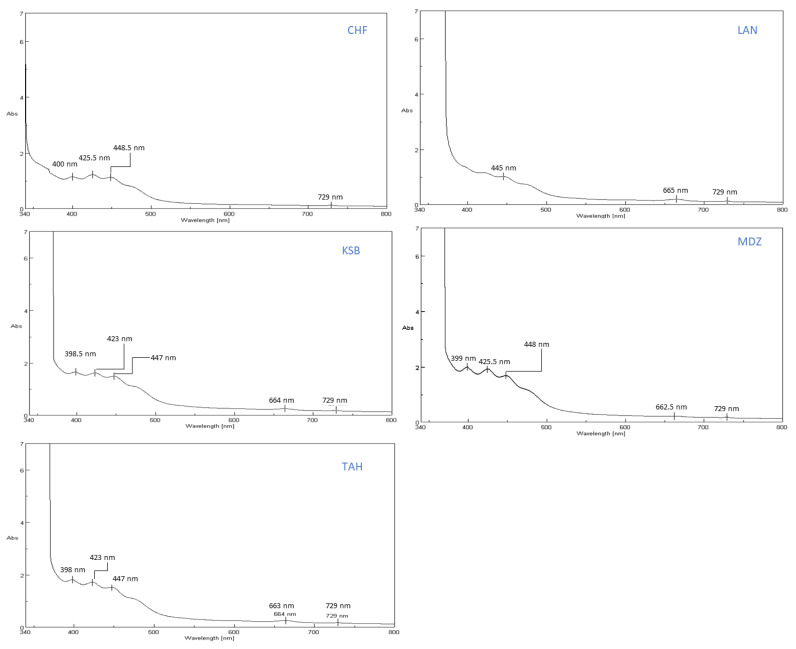
Ultra violet-visible spectroscopy analysis of strawberry tree fruits extracts.

The UV-VIS analysis of the samples extracts within the wavelength range of 340–800 nm, displayed significant difference in spectra profile of each samples, with different absorbance peaks revealed for each one (Figure 2). The CHF extract profile showed distinct absorbance peaks at 400, 425.5, 448.5, and 729 nm, with intensities of 1.142, 1.221, 1.127, and 0.130 respectively. The KSB extract exhibited several absorbance peaks at 398.5, 423, 447, 664, and 729 nm, with the absorption of 1.651, 1.612, 1.486, 0.247, and 0.187, respectively. Likewise, LAN sample extracts displayed peaks around 445, 665 and 729 nm, with the absorption of 1.009, 0.192, and 0.128, respectively. The MDZ extracts showed five peaks around 399, 424.5, 448, 662.5 and 729 nm, with the respective absorbances of 1.991, 1.919, 1.680, 0.212, and 0.176 (Figure 2). Finally, TAH extracts recorded distinct peaks at 398, 423, 447, 664, and 729 nm, with the absorption of 1.807, 1.709, 1.525, 0.254, and 0.181 respectively (Figure 2). Overall, owing to the UV-VIS spectra, fruit extracts of the examined cultivars displayed significant differences, mainly attributed to the phenotypic factor.

### 3.2. Biochemical Parameters

The results for titratable acidity, pH, and total soluble solids (TSS) in fruits for all genotypes are presented in Table 2. Analysis of the physicochemical data pertaining to the five genotypes showed significant variations in all parameters (*p* < 0.001).

The titratable acidity ranged from 0.65 to 1.01 g malic acid/100g FW, with an average of 0.83 g malic acid/100 g FW. The highest value was recorded in “TAH” (1.01 g malic acid/100 g FW) while the lowest value was observed in “MDZ” (0.65 g malic acid/100 g FW). The titratable acidity of strawberry tree fruits reported in this study was higher than those found by other authors, Ozan and Haciseferoğullari [42] and Vidrih et al. [31]. They found titratable acidity values of 0.51% and 0.40%, respectively. However, the results herein obtained were lower compared to the results reported by Doukani and Hadjer [43], who found (2.14%) in Algerian strawberry tree genotypes.

The pH values ranged from 2.44 “KSB” to 3.92 “LAN” with an average of 3.36. These results were approximately similar with those recorded by Ruiz Rodriguez et al. [8] and González et al. [44], who found 3.47 and 3.50, respectively. Nevertheless, the values obtained in this study were lower than those found by Serçe et al. [5] and Ozan and Haciseferoğullari [42], who found 5.57 and 4.6, respectively. 

The total soluble solids of the strawberry tree fruits varied from 14.83% “LAN” to 18.53% “KSB” with an average of 16.87%. Similar results were reported by Doukani and Tabak [45]. They found values comprised between 16.66 and 17.66%. The results obtained in this study were lower than those recorded by Celikel et al. [46] (21.4–30.0%) and by Vidrih et al. [31] (21.5%). The total soluble solids of strawberry tree fruits reported in this study were higher than those found by Muller et al. [47] and Serçe et al. [5], who found (8.1%) and (11.9%), respectively. The variations found may be due to different climatic conditions, region, and fruit ripeness [48,49].

### 3.3. Organic Acids and Ascorbic Acid 

The results for organic acids are summarized in Table 3. Highly significant variations were found at (*p* < 0.001) between genotypes. Four organic acids were identified by HPLC for all fruits of strawberry tree genotypes studied and citric acid was determined as the major organic acid in all genotypes, followed by malic acid, ascorbic acid, and succinic acid. The citric acid content ranged from 1.74 to 5.32 g/100 g with an overall mean of 3.12 g/100 g. Citric acid had the dominant presence in “LAN” (5.32 g/100 g), while its lowest amount was recorded in “KSB” (1.74 g/100 g). Malic acid ranged from 1.53 to 2.86 g/100 g with an overall average of 2.12 g/100 g. “KSB” also had the lowest malic acid content (1.53 g/100 g), with the highest value found in “TAH” (2.86 g/100 g). The ascorbic acid content was between 0.27 and 1.00 g/100 g with an overall average of 0.66 g/100 g. Ascorbic acid was significantly higher in “TAH” (10.02 g/100 g), while the lowest level was recorded by “KSB” (2.85 g/100 g). Succinic acid content ranged from 0.485 to 4.66 g/100 g with an overall average of 1.04 g/100 g. Succinic acid was significantly higher in “LAN” (4.66 g/100 g) while the lowest content was in “CHF” (0.485 g/100 g). Our results showed that “TAH” had the highest levels of malic acid and ascorbic acid while “KSB” had the lowest levels of malic acid and citric acid. 

The average citric acid content in our fruit was higher than that reported by Serçe et al. [5] and Doukani and Hadjer [43] who recorded 0.03 g/100 g and 8.56 mg/100 g respectively. However, Ruiz-Rodriguez et al. [8] showed a total absence of citric acid. In addition, the mean malic acid content in the fruits analyzed in this study was higher than those presented by Serçe et al. [5], and Doukani and Hadjer [43], who found proportions of 0.34 g/100 g and 282.3 mg/100 g, respectively. In addition, our results were also higher than that reported by Ayaz et al. [12], who reported malic acid content in fruits of a strawberry tree from Turkey (0.084 mg/100 g). On the other hand, our results were lower than those reported by Alarcão-E-Silva et al. [13], who showed a content of the order of (5.99 g/100 g) in fruits of strawberry tree from Portugal. In addition, mean ascorbic acid levels in our samples were higher than reported in Spanish strawberry tree fruits (6.03 mg/100 g) [7] and in Turkish strawberry tree fruits [46], where they ranged between 98.0 and 280.0 mg/100 g. Ascorbic acid was also reported to be present in fruits of strawberry tree, between 89–346 mg/100 g [13,50,51]. Comparing our results with those of other authors, we note the absence of some organic acids in our fruits, notably: oxalic, fumaric, lactic, suberic, and quinic acids. Indeed, fumaric (0.15 g/100 g), lactic (0.05 g/100 g), suberic (0.023 g/100 g), and quinic (7.35 g/100 g) acids were detected and quantified by Ayaz et al. [12] in fruits of a strawberry tree in the middle Black Sea region of Turkey. In Spain, the authors showed variable amounts of oxalic acid (0.05–0.15 g/100 g) [8,51]. In addition, our samples contained very high levels of succinic acid (0.39–4.66 g/100 g) in contrast to the results obtained by Doukani and Hadjer [43], who recorded traces of succinic acid in Algerian *A. unedo* fruits. The presence and composition of organic acids can be affected by various factors, such as growing conditions, maturity, season, geographical origin, and soil type.

### 3.4. Phytochemical Composition 

#### 3.4.1. Total Phenols (TP)

Total phenol contents of strawberry tree fruits are summarized in Table 4. Significant variation (*p* = 0.044) was observed among the genotypes. The total phenols ranged from 25.37 to 39.06 mg GAE/g DW, with an average of 30.98 mg/g DW. The highest value was recorded in “LAN” (39.06 mg/g DW) while the lowest value was recorded in “KSB” (25.37 mg/g DW). 

The TP of sampled fruits were higher than those reported by Doukani and Tabak [45]. Previous studies indicated a wide variation on total phenolic content among *A. unedo* genotypes, grown in diverse agro climatic conditions, including Spain, Croatia, and Turkey, which varied from 483 to 1973 mg GAE/100 g FW [8,20,31]. In another study, Seker and Toplu [52] reported a TPC variation from 17.7 to 25.8 mg GAE/g). Moreover, several studies [80.20] recorded TP values ranging from 483 and 627 mg GAE/100 g and from 951 to 1973 mg/100g in Turkish and Spanish strawberry tree genotypes respectively. Vidrih et al. [31] reported an average of 590 mg/100 g TP in Croatian strawberry tree fruits. According to these results, and despite natural variations, TP content in fruits of strawberry tree grown in Morocco fruits was always over 39.06 mg GAE/g DW, indicating that it could be considered an excellent source of polyphenols content, which is of great importance, in light of the fact that modern diets are often lacking in bioactive compounds.

#### 3.4.2. Total Flavonoids (TF) 

The results of the total flavonoids content are reported in Table 4. Significant differences in total flavonoids were observed at (*p* = 0.002) among genotypes. The total flavonoid content varied from 3.30 to 7.07 mg GAE/g DW, with an average of 5.20 mg GAE/g DW. The highest flavonoid content was observed in “TAH” (7.07 mg/g DW) and the lowest value was observed in “KSB” (3.30 mg/g DW). These concentrations are higher than those recorded by Jurica et al. [53] (0.23–0.28 mg EQ/g) and Bouzid et al. [54] (2014) (2.18–6.54 mg EC/g), and by Pallauf et al. [7] (0.32 mg/100 g edible portion). 

#### 3.4.3. Total Anthocyanins 

The total anthocyanins content is summarized in Table 4. Significant differences were found at (*p* = 0.024) among the genotypes. The anthocyanins quantity varied from 0.15 to 0.64 mg equivalent cya-3-glu/100g DW with an overall mean of 0.34 mg equivalent cya-3-glu/100 g DW. The highest total anthocyanins content was observed in “MDZ” (0.64 cya-3-glu/100 g DW), while the lowest was obtained by “KSB” (0.15 cya-3-glu/100 g DW). These values were lower than the ones reported by Pallauf et al. [7] (3.77 mg equivalent cya-3-glu/100 g DW). 

### 3.5. Antioxidant Activities 

The results obtained for antioxidant activity based on the radical scavenging capacity DPPH, ABTS, and β-carotene are reported in Table 5. Significant differences (*p* ˂ 0.001) were observed among the genotypes. The average antioxidant activities values were 8.93, 7.82, and 5.58 mg/g DW as determined by DPPH, ABTS, and β-carotene assays, respectively. 

The extracts of strawberry tree fruits had strong antioxidant capacity for β-carotene assay. The antioxidant potency, as determined by β-carotene assay, ranged from 1.08 to 13 AAE mg/g DW. Isbilir et al. [55] analyzed the bleaching activity of β-carotene. They found (0.185–0.317 AAE mg/mL) in Turkish strawberry tree fruits. 

All genotypes showed scavenging effects against DPPH radical ranging from 3.33 to 21.08 mg/g DW. Ben Salem et al. [35] showed that the value of scavenging activity in fruits of strawberry tree grown in Tunisia was (0.32 AAE mg/mL) and ranged from 0.278 to 0.589 AAE mg/mL.

The value of ABTS assay ranged from 2.25 to 19.58 mg ascorbic acid equivalent/g DW. Gündoğdu et al. [56] and Colak [20] recorded the antioxidant capacity (ABTS) values ranged between 17.51 and 30.06 µmol TE/g DW and between 18.07 and 33.41 μmol TE/g DW in Turkish *A. unedo* fruits. This difference was most probably due to differences in the extraction method and solvent used. The different antioxidant levels observed in this study may reflect a relative difference in the ability of antioxidant compounds in extracts to reduce the free radical DPPH, ABTS, and oxidative bleaching of β-carotene in vitro systems. Antioxidant activity was widely studied on strawberry tree fruits by using different antioxidant determining methods such as ABTS, TEAC, FRAP, DPPH, etc., and all studies indicated that *A. unedo* fruits had high antioxidant activity and antioxidant activity found to be genotype dependent. Moreover, the studies indicated that type of extraction of phenols present in fruits of *A. unedo* also influenced the antioxidant activity [7,8,14,39,51,52,53,55,56]. In addition, several reports showed that strawberry tree fruit is a highly potential antioxidant plant compared to other fruit, such as red and green grape and apples [57], pomace [58], pomegranate [59], grape [60,61], which can be linked to the high phenolic composition of strawberry tree fruits in polyphenols. 

### 3.6. Polyphenols Profiles 

Phenolic compounds contained in the studied strawberry tree fruits were analyzed using standard and sample solutions. Retention times and wavelength are presented in Table 6. 

A total of 17 phenolic compounds were identified in strawberry tree fruits. The results obtained were summarized in Table 7 and Table 8. Among the determined phenolic acids, gallic acid, catechin, chlorogenic acid, and ellagic acid was found to be the major phenolic acid (Table 6). Statistically significant differences were observed among genotypes for all phenolic compounds. 

Gallocatechol and catechin were the dominant compounds in all genotypes. The highest levels reported in “TAH” (65.31 mg/100 g DW) and “CHF” (49.36 mg/100 g DW) respectively and the lowest in levels in “CHF” (16.15 mg/100g DW) and “LAN” (22.09 mg/100 g DW) respectively. Gallic acid and Gallic acid derivatives were present in significantly higher amounts in “TAH” (36.93 mg/100 g DW) and (14.54 mg/100 g DW) respectively, the highest concentration of syringic acid was detected in “LAN” (7.94 mg/100g DW) and the lowest in “CHF” (4.27 mg/100g DW). Among the phenolic acid group, chlorogenic acid was detected in higher amounts in “TAH” (27.42 mg/100 g DW). 

Ellagic acid was also detected in all genotypes. The highest level was found in “TAH” (33.73 mg/100 g DW) and the lowest in “CHF” (8.42 mg/100 g DW). The minor compounds found in this study were rutin, cyanidine-3-5-diglucoside, and cyanidine-3-arabinoside. Rutin compounds, present in lower amounts in all genotypes. “LAN” had the highest quantity of rutin (1.26 mg/100 g DW) whereas the lowest amount was recorded in “TAH” (0.90 mg/100 g DW). 

Concerning the last two compounds, cyanidin 30.5 diglucoside and cyanidin 3 arabinoside, they were identified within only three genotypes (CHF, MDZ, and TAH). The lowest amounts of them recorded in “CHF” (0.61 mg/100g DW) and (0.36 mg/100g DW), respectively, whereas the largest ones were observed in “TAH” (3.30 mg/100 g DW) and (1.64 mg/100g DW), respectively.

Our results are consistent with those of Ganhão et al. [62] who had found catechin, gallic acid, ellagic acid, ellagic acid, chlorogenic acid, rutin, and cyanidin-3-glucoside in strawberry tree fruits collected in Spain. However, Ayaz et al. [12] reported that gallic acid (10.7 mg/g DW) was the main phenolic compound in strawberry tree fruits collected in Turkey, followed by protocatechuic acid, gentisic acid, p-hydroxybenzoic acid, vanillic acid and m-anisic acid. Distinctively, Mendes et al. [63] had identified other phenolic compounds in strawberry tree fruits collected in northeastern Portugal. These compounds were gallic acid glucoside, galloylquinic acid, quinic acid derivative, proanthocyanidin dimer, galloylshikimic acid, digalloylquinic acid, digalloyl shikimic acid, catechin monomer, proanthocyanidin trimer, strictinin ellagitannin, ellagitannin derivative, galloyl derivative, trigalloylshikimic acid, myricetin rhamnoside, quercetin glucoside, gallotannin, and ellagic acid rhamnoside.

### 3.7. Correlation among Variables

In order to identify the relations between biochemical traits, all variables were subjected to bivariate correlation using the Pearson coefficient. Significant correlations at the level of 0.05 or 0.01 are summarized in Table 9 and Table 10. In the current study, the correlation value was found between DPPH and total anthocyanins (r = 0.931 *). Similarly, links were noticed between pH and total phenols (r = 0.919 *) as well as between ABTS and both anthocyanins (r = 0.929 *) and DPPH (r = 1.000 **). Moreover, AA (β-carotene) was correlated to anthocyanins (r = 0.946 *), DPPH (r = 0.986 **) and ABTS (r = 0.989 **). The correlation between ellagic acid and each of the following parameters: gallic acid derivative, chlorogenic acid, ellagic acid derivative I, and ellagic acid derivative II were respectively 0.975 **, 0.968 **, 0.893 * and 0.953 *. The results obtained showed also, positive correlations between cyanidin−30.5-diglucoside and each of the following parameters: total flavonoids (r = 0.883 *)**,** moisture content (r = 0.894 *) and cyaniding-3-glucoside (r = 0.962 **). Importantly, our results have shown highly significant correlations between the TAA and DPPH results, TAA and TPC, also, between the DPPH and TPC, although the study was conducted only on five cultivars (Table 5). The TAC (beta-carotene) and TPC were not correlated, and no correlation was observed between the ABTS+ radical scavenging activity and beta-carotene values. In the same way, the study revealed links between cyanidin-3-arabinoside and total flavonoids (r = 0.896 *), cyaniding-3-glucoside (r = 0.994 **) and cyanidin−30.5-diglucoside (r = 0.986 **). Correspondingly, it conveyed correlations between chlorogenic acid and gallic acid derivative (r = 0.978 **) as well as between ellagic acid derivative I (r = 0.927 *). As far as gallocatechin concerned, the study portrayed a relationship between it and gallic acid (r = 0.992 **) and protocatechuic (r = 0.907 *). Equally, the results depicted connections between total soluble solids and both total phenols (r = −0.897 *) and pH (r = −0.912 *). They showed also ties between protocatechuic and both titratable acidity (r = 0.907 *) and gallic acid (r = 0.908 *) as well as between quercetin-3-glucoside and quercetin-3-xyloside (r = 0.913 *). Positive relations between the following variables were also manifested by the same study: cyanidin-3-glucoside and total flavonoids (r = 0.896 *). Catechin revealed negative links with gallic acid (r = −0.925 *) and gallocatechin (r = −0.926 *). Similarly, rutin had negative links with ellagic acid derivative I (r = −0.928 *). However, syringic acid showed positive correlations with gallocatechin (r = 0.886 *) and negative ones with catechin (r = −0.961 **). Likewise, ellagic acid derivative II conveyed positive connections with both gallic acid derivative (r = 0.968 **) and chlorogenic acid (r = 0.909 *). Correlation coefficients may provide information on the parameters that are potentially important in assessing strawberry tree genotypes [64]. Significant and strong correlated traits can be used to predict other ones, and could be considered of importance for genotypes characterization and discrimination [65].

### 3.8. Principal Component Analysis (PCA)

To achieve a better understanding of the trends and relationships among the many studied variables (32) for the different strawberry tree samples (5 genotypes), principal component analysis (PCA) based on correlation coefficients was used to discriminate between variables in the datasets (Table 11).

The aim of this analysis was to determine the main factors to reduce the number of effective parameters to use in classification of the strawberry tree genotypes based on their biochemical parameters. In our study, only a principal component loading of more than |0.5| was considered as being significant for each factor. The first three components consisted of 32 variables, which explained 90.25% of the total variability observed (Table 11), which means that these characters had the highest variation between the genotypes and had the highest impact on discrimination of them. The first component accounted for 41.47% of the total variance, which is strongly influenced by the total flavonoids (0.72), anthocyanins (0.51), AA β-carotene (0.58), protocatechuic (0.59), gallic acid derivative (0.89), chlorogenic acid (0.91), ellagic acid derivative I (0.91), ellagic acid derivative II (0.83), ellagic acid (0.96), cyanidin-3-glucoside (0.84), rutin (−0.90), cyanidin-30.5-diglucoside (0.92), and cyanidin-3-arabinoside (0.89). The second component accounted for 32.04% of the total variance and is mainly influenced by anthocyanins (−0.83), DPPH (−0.75), ABTS (−0.74), β-carotene (−0.72), titratable acidity (0.60), gallic acid (0.84), protocatechuic (0.66), gallocatechin (0.84), catechin (−0.68), syringic acid (0.70), quercetin-3-xyloside (0.87), and quercetin-3-glucoside. The third component represents 16.74% of the total variation, which is defined essentially by total phenols (0.81), total flavonoids (0.60), titratable acidity (0.60), pH (0.84), total soluble solids (−0.87), and catechin (−0.51). Generally, these results are in accordance with those reported in previous strawberry tree biochemical studies [20,55]. They have reported that the biochemical attributes are important in order to evaluate the variation in traits of strawberry tree genotypes. These parameters can be used as a useful tool for selecting genotypes for breeding programs, or to recommend new cultivars with superior traits.

**Figure 3:**
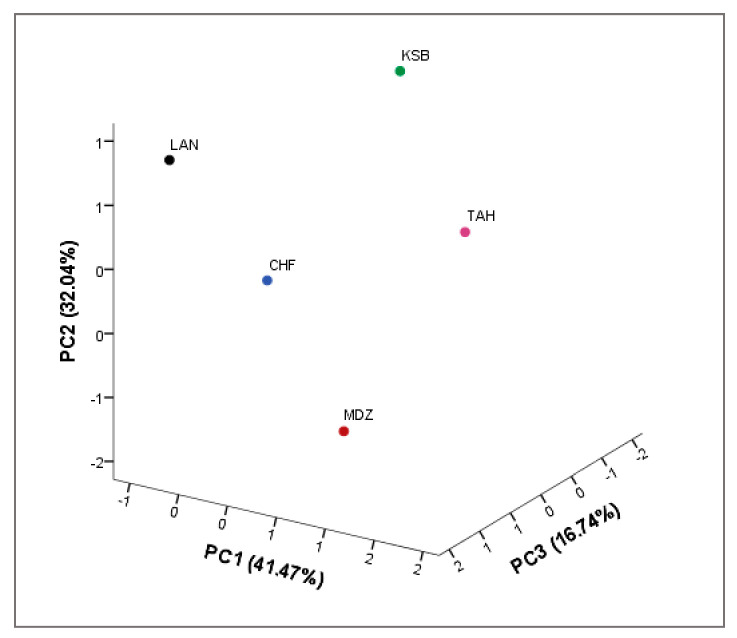
Scatter plot for the first three principal components (PC1/PC2/PC3, 89.79% of total variance) for the studied strawberry tree genotypes based on their physicochemical and biochemical parameter.

A three-dimensional (3D) scatter plot was prepared according to the first three principal components: PC1, PC2, and PC3, (respectively 41.47, 32.04, and 16.74% of total variance), that discriminate between the genotypes according to their physicochemical and biochemical characteristics (Figure 3). Starting from negative to positive values of PC1, the distribution of genotypes indicated an increased in the moisture content, total soluble solids, and the most of phenolic compounds. Whereas, starting from negative to positive values of PC2, total phenols, total flavonoids, and total anthocyanins decreased in their values. However, starting from negative to positive values of PC3, the distribution of genotypes indicated an increase in the pH, titratable acidity, and antioxidant activity (DPPH, ABTS, and β-carotene). Cultivar distribution showed a very distinctive profile of each sampled cultivar, since each one formed a single sub-cluster. This indicates a potential phenotypic divergence within the species, which deserves to be further explored in the agroecosystems prospected, in order to build a collection to preserve these resources vital to the advancement of the local agricultural and livelihood. Overall, our results are in agreement with several studies [20,55]. These studies indicated that high diversity in biochemical traits could be used as an efficient marker system to discriminate between strawberry tree genotypes.

### 3.9. Two Dimensional Clustered Heatmap 

**Figure 4:**
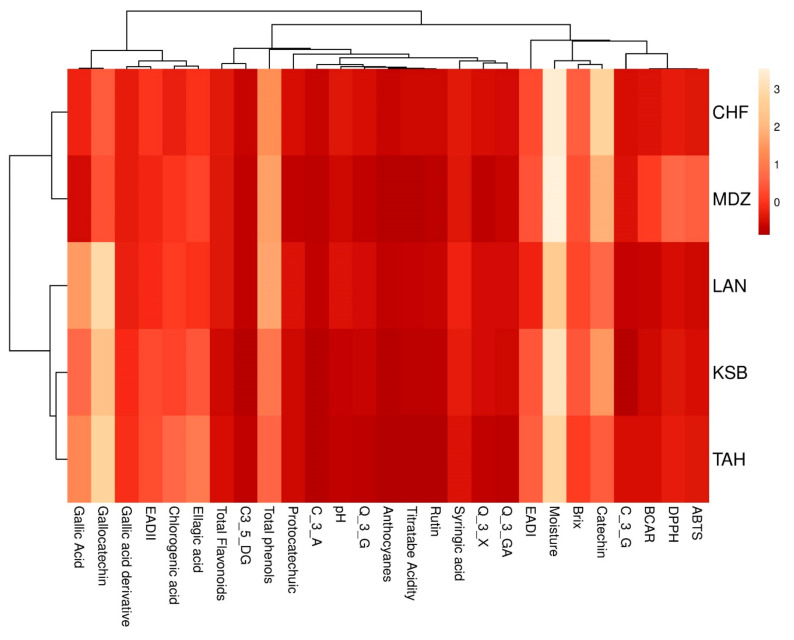
Two-dimensional hierarchically clustered heatmap based on the correlation distance of physicochemical and biochemical traits of fruit.

A hierarchically clustered heatmap is one of numerous analyses that does not require a dimensionality reduction to visualize dataset distribution. It is a widely used technique to analyze complex biological data by displaying network connections in a symmetric adjacency matrix. It was performed to obtain a simplified representation of the fruit physico-biochemical diversity within the dataset of sampled strawberry trees. A color-coded two-dimensional heatmap for both fruit parts are formed with two clusters using Euclidean distance following the Ward method; one is sample-oriented, whereas the other is variable-oriented (Figure 4). Figure 4 displays a colored data matrix, which gives an overview of the numeric differences between studied samples. In this figure, strong effect on the dataset is displayed in low yellow color intensity, while the weak one is shown with a high intensity red color. The heatmap showed that the moisture content had the higher scores in the dataset, followed, order of importance, by total phenols, gallocatechin and catechin, which means that these variables had the higher effect in cultivar clustering. However, the other variables showed a very weak impact on the dataset distribution. The cultivars were clustered based on their similarity into two groups. The first one included CHF and MDZ. On the other hand, the cultivars LAN, KSB and TAH, were classified as a single subset. The slightest divergence between PCA and heatmap can be referred to the amount of variability expressed in each method. The two-dimensional heatmap considers the entire characterization data and incriminates the whole variability, while the total inertia explained by the first three principal components of the scatter plot, was relatively reduced [66]. 

Data visualization is an essential tool for biochemical data analysis, and dimensionality reduction methods. Principal component analysis (PCA) is usually used to draw high dimensional data onto two- or three-dimensional space so it can be visualized. However, this transition is costly, often resulting in loss of the total variance. In the opposite, the hierarchically clustered heatmaps do not need a dimensionality reduction to visualize complex biological data by displaying network connections in a symmetric adjacency matrix. The two abovementioned chemometric approaches were widely used in several studies to discriminate biological material, such as apple juice [67,68], litchi fruits [69], and Surinam cherry leaves [70].

## 4. Conclusions

This study is likely to provide the first set of data on the physicobiochemical attributes of strawberry tree fruits (*Arbutus unedo*) prospected in an endemic area, where this species is spontaneously growing in Morocco. Results displayed significant differences among sampled trees based on the investigated physicobiochemical attributes. Thus, titratable acidity was in the range of 0.65 and 1.01 g malic acid/100g FW, whereas, total soluble solids varied from 14.83% to 18.53%. Citric acid was the major organic acid, followed by malic acid, where the average concentrations ranged from 1.74 to 5.32 g/100 g and from 1.53 to 2.86 g/100 g, respectively. Results also showed that the strawberry tree fruits could be considered as interesting, high-value nutraceuticals, being a novel source of bioactive compounds for dietary supplements or functional foods. Indeed, total phenols ranged from 25.37 to 39.06 mg GAE/g DW, while total flavonoid content varied between 3.30 and 7.07 mg GAE/g DW. Seventeen phenolic compounds were identified by HPLC, of which gallocatechol and catechin were the most abundant, of which the highest level was reported in “TAH” (65.31 mg/100 g DW). Given the current day, the biochemical composition of the strawberry tree fruits could be useful to improve future pharmacological and cosmetic usages. In addition, the results found in this study may be helpful for nutritionists, as well as berry growers and breeders, who can promote the cultivation of species and new cultivars with higher phenolic content and antioxidant activity. The high variability in biochemical composition observed among genotypes could be attributed to genetic factors. Therefore, it will be important to study and identify the genes responsible for the biochemical properties in order to understand the pattern of variation in the biochemical composition of strawberry tree genotypes. The present work provides important data for the food and pharmaceutical industries to consider this fruit as an exotic (or unusual) source of bioactive compounds, colors, and flavors.

## Figures and Tables

**Table 1:** Geographic origin of the strawberry tree genotypes studied.

Origin	Code	Zone	Altitude (m)	Longitude (E)	Latitude (N)
Chefchaouen	CHF	Rif	534	5°17′07″	35°07′41″
Moulay Driss Zerhoun	MDZ	Middle Atlas	820	5°30′26″	34°02′33″
Laanoucer	LAN	Middle Atlas	1700	4°54′39″	33°42′06″
El Ksiba	KSB	Middle Atlas	1360	6°01’23″	32°31’36″
Tahnaout	TAH	High Atlas	1200	7°55′07″	31°18′14″

**Table 2:** Physicochemical parameters in fruits of the strawberry tree genotypes.

Genotype Name	TA (g Malic Acid/100 g FW)	pH	TSS (%)
KSB	0.72 ± 0.02 a,b	2.44 ± 0.03 a	18.53 ± 0.50 d
CHF	0.81 ± 0.01 b	3.76 ± 0.01 c	16.63 ± 0.40 b
MDZ	0.65 ± 0.01 a	3.71 ± 0.01 c	16.83 ± 0.29 b,c
LAN	0.97 ± 0.01 c	3.92 ± 0.02 d	14.83 ± 0.29 a
TAH	1.01 ± 0.10 c	2.99 ± 0.10 b	17.53 ± 0.45 c
Mean	0.83	3.36	16.87
Std. Deviation	0.15	0.58	1.30
ANOVA Mean square	0.07 ***	1.19 ***	5.56 ***

*** denote significant of difference at level 0.001; data values are means ± SD; values in bold represent, in each column, the minimum and the maximum for each variable; Different letters (a–d) in the columns represent statistically significant differences among genotypes according to Duncan’s multi-range test at *p* ˂ 0.05; TA: titratable acidity; Fresh weight (FW); TSS: total soluble solids.

**Table 3:** Composition of organic acids and ascorbic acid (g/100 g DW) in fruits of strawberry tree genotypes.

Genotype Name	Citric Acid	Malic Acid	Ascorbic Acid	Succinic Acid
CHF	32.24 ± 1.06 c,d	23.58 ± 0.84 e	7.05 ± 0.89 c	4.85 ± 0.38 a,b
KSB	17.40 ± 3.16 a	15.27 ± 2.92 a	2.85 ± 0.76 a	5.98 ± 1.35 b
MDZ	27.62 ± 1.04 b,c	18.85 ± 0.78 a,b,c	9.49 ± 0.66 f	7.69 ± 0.56 c
LAN	53.23 ± 4.07 e	23.15 ± 1.50 d,e	6.80 ± 0.38 c	46.60 ± 1.21 f
TAH	28.00 ± 1.49 b,c	28.65 ± 1.24 f	10.02 ± 0.16 f	11.07 ± 0.19 d

Values in bold are minimum and maximum; different letters (a–g) in the columns represent statistically significant differences between genotypes, according to Duncan’s multi-range test at *p* ˂ 0.001, DW (dry weight)

**Table 4:** Phytochemical composition (total phenols, total flavonoids, total anthocyanins) in fruits of strawberry tree genotypes.

Genotype Name	Total Phenols (mg GAE/g DW)	Total Flavonoids (mg RE/g DW)	Total Anthocyanins (mg C-3-GE/100g DW)
KSB	25.37 ± 5.60 a	3.30 ± 0.60 a	0.15 ± 0.09 a
CHF	28.71 ± 7.34 a	4.49 ± 0.87 a,b	0.30 ± 0.14 a
MDZ	34.72 ± 6.53 a,b	6.09 ± 0.88 c,d	0.64 ± 0.20 b
LAN	39.06 ± 2.44 b	5.07 ± 1.04 b,c	0.18 ± 0.09 a
TAH	27.07 ± 0.96 a	7.07 ± 0.67 d	0.43 ± 0.23 a,b
Mean	30.98	5.20	0.34
Std. deviation	6.88	1.51	0.23
ANOVA Mean square	98.39 *	6.31 **	0.12 *

* Denote significant of difference at level 0.05; ** denote significant of difference at level 0.01; data values are means ± SD; values in bold represent, in each column, the minimum and the maximum for each variable; different letters (a–d) in the columns represent statistically significant differences among genotypes according to Duncan’s multi-range test at *p* ˂ 0.05; GAE: gallic acid equivalent; RE: rutin equivalent; C-3-GE: cyanidin-3-glucoside equivalent.

**Table 5:** Free radical scavenging activity assessed by DPPH, ABTS, and β-Carotene blanching essay investigated in fruits of sampled strawberry tree genotypes. Results are expressed as mean ± SD in mg Ascorbic acid equivalent (AAE)/g DW.

Genotype Name	DPPH	ABTS	β-CAROTENE
KSB	5.75 ± 2.00 a,b	4.83 ± 1.88 a,b	3.50 ± 0.75 a,b
CHF	4.50 ± 2.41 a,b	3.33 ± 1.13 a	2.83 ± 0.76 a
MDZ	21.08 ± 5.55 c	19.58 ± 4.49 c	13.00 ± 4.34 c
LAN	3.33 ± 1.51 a	2.25 ± 0.90 a	1.08 ± 0.38 a
TAH	10.00 ± 3.77 b	9.08 ± 3.01 b	7.50 ± 3.12 b
Mean	8.93	7.82	5.58
Std. deviation	7.29	6.92	4.87
ANOVA Mean square	157.43 ***	150.03 ***	68.12 ***

*** Denote significant of difference at level 0.001; values in bold represent, in each column, the minimum and the maximum for each variable; different letters (a–c) in the columns represent statistically significant differences among genotypes according to Duncan’s multi-range test at *p* ˂ 0.05, AAE (Ascorbic acid equivalent)

**Table 6:** Retention time and wavelength of phenolic compounds at Lambda 280 nm, Lambda 360 nm, and Lambda 520 nm.

Phenolic Compounds	Retention Times (min)	Wavelength (nm)
Gallic acid	7.25	280
Protocatechuic	9.18	280
Gallocatechin	10.39	280
Gallic acid derivative	13.35	280
Catechin	14.97	280
Chlorogenic acid	16.38	280
Syringic acid	16.81	280
Ellagic acid derivative i	19.67	280
Ellagic acid derivative ii	21.22	280
Ellagic acid	23.33	280
Quercetin-3-xyloside	21.26	360
Rutin	22.81	360
Quercetin-3-galactoside	25.67	360
Quercetin-3-glucoside	26.01	360
Cyanidin-30.5-diglucoside	14.10	520
Cyanidin-3-glucoside	14.59	520
Cyanidin-3-arabinoside	16.09	520

**Table 7:** Polyphenolic compounds at genotypes site (mean ± SD in mg/100 DW).

Genotype Name	GA	PC	GC	GAD	CAT	CA	SA	EADI
**KSB**	21.88 ± 0.01 c	3.14 ± 0.01 c	45.23 ± 0.05 c	10.15 ± 0.01 d	33.60 ± 0.03 c	14.50 ± 0.00 d	7.40 ± 0.01 c	18.9 ± 0.01 d
**CHF**	6.09 ± 0.00 b	2.57 ± 0.01 b	**16.15 ± 0.03 a**	**4.98 ± 0.00 a**	**49.36 ± 0.01 e**	**5.55 ± 0.00 a**	**4.27 ± 0.00 a**	13.32 ± 0.01 b
**MDZ**	**4.56 ± 0.02 a**	**1.84 ± 0.00 a**	17.11 ± 0.07 b	7.36 ± 0.01 c	38.98 ± 0.05 d	12.10 ± 0.01 b	6.17 ± 0.01 b	17.22 ± 0.05 c
**LAN**	35.83 ± 0.02 d	4.18 ± 0.03 d	58.79 ± 0.33 d	7.30 ± 0.01 b	22.09 ± 0.08 a	12.48 ± 0.02 c	**7.94+ ± 0.02 e**	**8.05 ± 0.03 a**
**TAH**	**36.93 ± 0.02 e**	**5.90 ± 0.01 e**	**65.31 ± 0.04 e**	**14.54 ± 0.02 e**	24.68 ± 0.08 b	**27.42 ± 0.02 e**	7.80 ± 0.01 d	**25.06 ± 0.04 e**
**Mean**	21.06	3.53	40.52	8.87	33.74	14.41	6.72	16.45
**Std. deviation**	14.40	1.46	21.27	3.39	10.24	7.42	1.42	5.85
**ANOVA**	725.36 ***	7.49 ***	1584.06 ***	40.19 ***	327.11 ***	192.58 ***	7.06 ***	119.70 ***
**Mean square**								

*** denote significant of difference at level 0.001; data values are means ± SD; values in bold represent, in each column, the minimum and the maximum for each variable; different letters (a–e) in the columns represent statistically significant differences among genotypes according to Duncan’s multi-range test at *p* ˂ 0.05.

**Table 8:** Polyphenolic compounds at genotypes site (mean ± SD in mg/100 DW).

Genotype name	EADII	EA	C3G	Q3X	RT	Q3G	Q3G	C3.5DG	C3A
**KSB**	15.96 ± 0.01 c	18.00 ± 0.00 d	**0.43 ± 0.01 a**	**4.09 ± 0.01 e**	1.06 ± 0.01 c	**3.46 ± 0.02 d**	**2.89 ± 0.00 d**	n.d	n.d
**CHF**	**8.97 ± 0.01 a**	**8.42 ± 0.01 a**	2.27 ± 0.00 c	2.11 ± 0.01 b	1.17 ± 0.00 d	**1.66 ± 0.00 a**	**2.11 ± 0.01 a**	**0.61 ± 0.00 a**	**0.36 ± 0.01 a**
**MDZ**	9.40 ± 0.04 b	14.34 ± 0.02 c	5.68 ± 0.01 d	**1.43 ± 0.01 a**	0.96 ± 0.00 b	3.02 ± 0.01 c	2.12 ± 0.01 a	1.59 ± 0.02 b	1.07 ± 0.00 b
**LAN**	9.40 ± 0.10 b	10.27 ± 0.05 b	0.57 ± 0.02 b	2.72 ± 0.03 c	1.26 ± 0.01 e	3.03 ± 0.04 c	2.54 ± 0.02 c	n.d	n.d
**TAH**	**21.39 ± 0.02 d**	**33.73 ± 0.02 e**	**7.21 ± 0.01 e**	2.81 ± 0.03d	**0.90 ± 0.02 a**	2.73 ± 0.02 b	2.27 ± 0.01 b	**3.30 ± 0.02 c**	**1.64 ± 0.01 c**
**Mean**	13.02	16.95	3.23	2.63	1.07	2.78	2.39	1.10	0.61
**Std. deviation**	5.10	9.34	2.84	0.91	0.14	0.63	0.30	1.29	0.67
**ANOVA**	90.92 ***	305.06 ***	28.25 ***	2.91 ***	0.06 ***	1.38 ***	0.33 ***	5.82 ***	1.55 ***
**Mean square**									

*** denote significant of difference at level 0.001; data values are means ± SD; values in bold represent, in each column, the minimum and the maximum for each variable; different letters (a–e) in the columns represent statistically significant differences among genotypes according to Duncan’s multi-range test at *p* ˂ 0.05.

**Table 9:** Correlation coefficients among biochemical parameters analyzed.

	TP	TF	ANT	DPPH	ABTS	BCAR	TA	PH	TSS	GA	PC	GC	GAD	CAT	CA	SA	EADI	EADII	EA	C3G	Q3X
**TP**	1																				
**TF**	0.218	1																			
**ANT**	0.138	0.725	1																		
**DPPH**	0.144	0.540	0.931 *	1																	
**ABTS**	0.130	0.545	0.929 *	1.000 **	1																
**BCAR**	0.017	0.602	0.946 *	0.986 **	0.989 **	1															
**TA**	0.103	0.416	−0.316	−0.524	−0.516	−0.453	1														
**PH**	0.919*	0.378	0.331	0.211	0.194	0.112	0.119	1													
**TSS**	0.897*	−0.194	0.062	0.188	0.204	0.283	−0.383	−0.912 *	1												
**GA**	0.070	0.245	−0.436	−0.449	−0.436	−0.401	0.844	−0.118	−0.166	1											
**PC**	−0.164	0.467	−0.224	−0.352	−0.336	−0.247	0.907 *	−0.202	−0.014	0.908 *	1										
**GC**	−0.015	0.236	−0.413	−0.401	−0.385	−0.345	0.792	−0.217	−0.050	0.992 **	0.907*	1									
**GAD**	−0.464	0.460	0.076	0.092	0.114	0.212	0.455	−0.547	0.505	0.662	0.786	0.736	1								
**CAT**	−0.291	−0.335	0.228	0.157	0.145	0.148	−0.659	−0.039	0.226	−0.925 *	−0.756	−0.926 *	−0.628	1							
**CA**	−0.302	0.615	0.175	0.154	0.175	0.267	0.542	−0.364	0.341	0.700	0.829	0.757	0.978 **	−0.694	1						
**SA**	0.151	0.204	−0.239	−0.083	−0.068	−0.075	0.470	−0.156	−0.005	0.854	0.658	0.886 *	0.687	−0.961**	0.707	1					
**EADI**	−0.716	0.436	0.407	0.397	0.416	0.533	0.027	−0.645	0.788	0.124	0.396	0.221	0.819	−0.091	0.763	0.201	1				
**EADII**	−0.656	0.318	−0.034	−0.039	−0.016	0.100	0.426	−0.703	0.630	0.590	0.757	0.669	0.968 **	−0.478	0.909 *	0.549	0.853	1			
**EA**	−0.501	0.580	0.239	0.199	0.220	0.334	0.426	−0.501	0.521	0.541	0.747	0.612	0.975 **	−0.496	0.968 **	0.534	0.893 *	0.953 *	1		
**C3G**	−0.143	0.896 *	0.847	0.698	0.706	0.790	0.118	0.064	0.212	−0.024	0.289	0.010	0.516	−0.050	0.605	0.009	0.716	0.440	0.674	1	
**Q3X**	−0.509	−0.553	−0.777	−0.604	−0.590	−0.564	0.197	−0.769	0.472	0.537	0.390	0.592	0.455	−0.395	0.305	0.532	0.186	0.549	0.289	−0.501	1
**RT**	0.470	−0.578	−0.690	−0.709	−0.724	−0.807	0.161	0.394	−0.661	0.054	−0.191	−0.039	−0.685	0.043	−0.673	−0.152	−0.928 *	−0.650	−0.782	−0.842	0.102
**Q3GA**	0.119	−0.105	−0.103	0.218	0.229	0.173	−0.155	−0.247	0.228	0.385	0.104	0.452	0.421	−0.627	0.382	0.798	0.153	0.291	0.261	−0.132	0.484
**Q3G**	−0.202	−0.646	−0.747	−0.474	−0.465	−0.499	0.000	−0.557	0.297	0.449	0.157	0.497	0.252	−0.440	0.121	0.600	−0.062	0.280	0.046	−0.655	0.913*
**C3.5D**	−0.263	0.883 *	0.671	0.499	0.512	0.620	0.329	0.197	0.521	0.541	0.747	0.232	0.685	−0.195	0.759	0.143	0.789	0.632	0.820	0.962**	−0.300
**C3A**	−0.197	0.896 *	0.785	0.630	0.641	0.735	0.196	0.001	0.242	0.064	0.382	0.100	0.594	−0.111	0.676	0.068	0.758	0.526	0.743	0.994**	−0.419
**C AC**	0.833	0.176	−0.217	−0.342	−0.354	−0.421	0.552	0.806	−0.974 **	0.373	0.207	0.261	−0.357	−0.390	−0.204	0.166	−0.743	−0.475	−0.403	−0.262	−0.288
**M AC**	0.049	0.718	0.168	−0.158	−0.154	−0.063	0.827	0.272	−0.340	0.460	0.716	0.405	0.347	−0.287	0.472	0.061	0.201	0.328	0.446	0.529	−0.279
**A AC**	0.292	0.955 *	0.805	0.578	0.577	0.622	0.283	0.530	−0.297	−0.002	0.246	−0.030	0.196	−0.084	0.364	−0.073	0.286	0.073	0.355	0.866	−0.765
**S AC**	0.782	0.062	−0.386	−0.383	−0.389	−0.466	0.559	0.595	−0.823	0.609	0.330	0.526	−0.128	−0.675	−0.011	0.526	−0.654	−0.272	−0.253	−0.381	0.042

* Correlation is significant at the 0.05 level; ** correlation is significant at the 0.01 level; TP: total phenols; TF: total flavonoids; ANT: anthocyanins; βCAR: β-carotene; TA: titratable acidity; TSS: total soluble solids; GA: gallic acid; PC: protocatechuic; GC: gallocatechin; GAD: gallic acid derivative; CAT: catechin; CA: chlorogenic acid; SA: syringic acid; EADI: ellagic acid derivative I; EADII: ellagic acid derivative II; EA: ellagic acid; C3G: cyanidin-3-glucoside; Q3X: quercetin-3-xyloside.

**Table 10:** Correlation coefficients among biochemical parameters analyzed.

	RT	Q3GA	Q3G	C3,5D	C3A	C AC	M AC	A AC	S AC
RT	1								
Q3GA	−0.220	1							
Q3G	0.237	**0.705**	1						
C3,5D	**−0.822**	−0.124	**−0.529**	1					
C3A	**−0.849**	−0.119	**−0.603**	0.986 **	1				
C AC	**0.684**	−0.160	−0.152	−0.250	−0.269	1			
M AC	−0.112	**−0.519**	**−0.539**	**0.653**	**0.574**	0.410	1		
A AC	−0.471	−0.321	**−0.839**	**0.799**	**0.842**	0.224	**0.695**	1	
S AC	**0.627**	0.241	0.228	−0.326	−0.367	0.907 *	0.217	0.001	1

* Correlation is significant at the 0.05 level; ** correlation is significant at the 0.01 level; RT: rutin; Q3GA: quercetin-3-galactoside; Q3G: quercetin-3-glucoside; C3.5D: cyanidin-3.5-diglucoside; C3A: cyanidin-3-arabinoside; C AC: Citrique acid; M AC: Malique acid; A AC: Ascorbic acid; S AC: Succinic acid.

**Table 11:** Eigenvectors of principal component axes from principal component analysis (PCA) analysis of studied variables.

Variables	Component			
	1	2	3	4
Total phenols	−0.364	−0.138	**0.784**	0.483
Total flavonoids	**0.773**	−0.216	**0.596**	0.006
Anthocyanins	**0.576**	**−0.772**	0.188	0.195
DPPH	**0.504**	**−0.708**	−0.010	0.494
ABTS	**0.522**	**−0.695**	−0.019	0.494
Β-carotene	**0.625**	**−0.673**	−0.048	0.393
Titratable acidity	0.263	**0.657**	**0.599**	−0.374
pH	−0.283	−0.384	**0.860**	0.183
Soluble solids	0.443	0.015	**−0.894**	−0.068
Gallic acid	0.299	**0.888**	0.345	0.064
Protocatechuic	**0.559**	**0.726**	0.323	−0.237
Gallocatechin	0.365	**0.893**	0.245	0.100
Gallic acid derivative	**0.846**	**0.512**	−0.141	0.053
Catechin	−0.332	**−0.745**	−0.398	−0.419
Chlorogenic acid	**0.881**	0.464	0.057	0.078
Syringic acid	0.352	**0.746**	0.151	**0.545**
Ellagic acid derivative I	**0.898**	0.011	−0.428	−0.107
Ellagic acid derivative II	**0.787**	**0.525**	−0.296	−0.136
Ellagic acid	**0.931**	0.342	−0.110	−0.059
Cyanidin-3-glucoside	**0.891**	−0.401	0.203	−0.067
Quercetine-3-Xyloside	−0.060	**0.835**	**−0.546**	0.024
Rutin	**−0.908**	0.259	0.303	−0.131
Quercetin-3-galactoside	0.156	0.411	−0.237	**0.866**
Quercetin-3-glucoside	−0.260	**0.752**	−0.477	0.373
Cyanidin-30.5-diglucoside	**0.950**	−0.160	0.192	−0.185
Cyanidin-3-arabinoside	**0.926**	−0.307	0.191	−0.106
citric acid	−0.402	0.208	**0.891**	0.033
Malic acid	0.467	0.147	**0.658**	**−0.573**
Ascorbic acid	**0.617**	−0.451	**0.638**	−0.093
Succinic acid	−0.348	**0.496**	**0.734**	0.306
% of variance	41.47	32.04	16.74	9.75
Cumulative %	41.47	73.51	90.25	100.00
